# Complexity theory for the modern Chinese economy from an information entropy perspective: Modeling of economic efficiency and growth potential

**DOI:** 10.1371/journal.pone.0227206

**Published:** 2020-01-28

**Authors:** Jun Yan, Lianyong Feng, Artem Denisov, Alina Steblyanskaya, Jan-Pieter Oosterom

**Affiliations:** 1 School of Economics and Management, China University of Petroleum (Beijing), Beijing, China; 2 Department of Computer Science, Kostroma State University, Kostroma, Russia; 3 School of Economics and Management, Harbin Engineering University, Harbin, China; 4 Laboratory of Microanalysis and Modeling, Economics and Mathematics Institute, Russian Academy of Science, Moscow, Russia; 5 Department of Management Accounting and Control, Nyenrode Business University, Breukelen, Netherlands; Shandong University of Science and Technology, CHINA

## Abstract

Complexity modelling of economic efficiency and growth potential is increasingly essential for countries and provinces. Evaluating the monetary flows, kinetic energy (efficiency) and potential capacity (resilience) provides crucial information for economic development. In the paper, the authors analyze growth opportunities for the Chinese economy from a system science point of view, using the perspective of information entropy, based on the input-output tables. Over the past four decades of reform and opening-up, China has made remarkable progress in its economic development. In 2007, China’s GDP was at its fastest pace in history at 14.2% growth. However, after the financial crisis in 2008, the global economy experienced a downward trend and China's economic development also settled on a medium-low level of development. The traditional perspective is to rank regional development only based on GDP growth, whereas here, the authors advocate another evaluation method based on efficiency and potential growth. Unbalanced regional economic development has become problematic and has become a barrier for sustainability of China’s economy. The results of the research indicate firstly that China’s regional development in 2007 and 2012 has been unequal between the provinces. Secondly, the authors found that Shandong province had significantly higher indicators for efficiency and potential growth than others in the same circumstances. Authors observe that provinces tend to carry out industrial policies and adjust the structure of industry on a local level. This analysis demonstrates that the spatial imbalance of efficiency and potential of economic development under the perspective of provincial-level regions. From the perspective of industry, it indicates that the supply chain is too short, mainly focusing on the mining and processing of resources and minerals in the original upstream industry chain, while the downstream is not fully utilized. These represent some unique insights yielded through this type of analysis that authors advocate applying more broadly.

## Introduction

In the paper, the authors pursue the goal of comparing China’s provinces and industries in order to identify which one is the most efficiency and has the highest potential growth. What are the characteristics of economic development? Can the parameters of efficiency and resilience reflect the underlying economic characteristics? The authors try to analyze Chinese economy by using a system science approach, complex science methodology.

After the founding of the People's Republic of China in 1949, the Chinese people have focused on survival. Early reform and opening up started in 1978, followed by finding a better way to develop the economy and exploring path of sustainable development. The characters of typical businesses were the processing with supplied materials, processing with supplied samples, assembling with supplied pieces and compensation trade, cheap labour, processed for exports. In 1992, the real estate reform started and Shenzhen city as one of the individual zones, taking the lead in industrial modernization. Overcoming the impact of severe floods along the Yangtze River in 1998 and the SARS virus in 2003, China’s economy developed rapidly, evidenced by housing prices that skyrocketed and infrastructure building to welcome the Olympic Games in 2008. The CPC (central government) had boosted domestic demand by 4 trillion RMB to reduce the impact of the global financial crisis. This slowed the recession[1]. Currently, China is promoting supply-side reforms, high-quality economic development, structural adjustments and steady growth[2]. The potential capacity of the Chinese economy has provided ample room to meet external challenges and achieve high-quality development[3]. The Chinese government keeps the economy running within a certain range and believes the Chinese economy is strongly resilient[4]. The Authors try to analyze Chinese Economy by using historical input-output data.

Thus, input and output analysis (referred to as IOA) can reflect the input-output relationship between various areas of the economic system comprehensively and systematically, and reveal the inner economic relations of mutual dependence and mutual restriction between different industries in the production process. The function of input-output accounting is not only to reflect the direct and obvious economic links between various sectors in the production process, but also to reveal the indirect, hidden links between various sectors. It provides a basis for studying the industrial structure and carrying out multiple quantitative analysis. Leontief began studying input-output technology and compiling input-output tables in 1931[5]. The construction and usage of Input-Output Tables in China economic analysis study began in 1960. Chen Xikang and his students, constructed the first experimental input-output table of the national economy of China for 1973. Since the reform and opening to the outside world (1978), major changes have taken place in China. Input-output analysis has been applied not only in macroeconomic analysis, but also in microeconomic management[6]. With the rapid spread of the input-output technique in China, it is necessary to now exchange experience and search together for solutions to the problems in theory and its applications. After the third plenary session of the 11^th^ CPC central committee, China focused on economic construction, which created the conditions for the research and application of modern economic analysis methods including input-output techniques. Since then, input-output table preparation, input-output technology research and input-output table application has rapidly developed[7]. In the 1960s, Romania's famous economist Nicholas Georgescu—Roegen (1906–1994), first introduced the concept of entropy from physics to economics. He published a book on the laws of entropy and the economic process, illustrating how the essential means of economic activity continued to increase entropy[8]. In 1997, professor Wang Hengjun of Xiaan university of Posts and Telecommunications in China also advocated applying the concepts of energy and entropy in physics into economics and put forward the concepts of economic energy, economic entropy, absolute economic entropy, and relative economy. He believed that entropy caused economic crises, which was an inevitable social and economic phenomenon[9]. In the USA, Yakovenko, the professor of physics at the University of Maryland, found that economic systems and thermal systems are analogous. Thus, Yakovenko creatively introduced the entropy principle in the economic research field in 2000, pointing out that money is similar to the of conservation of energy. That means that in a closed economy, the monetary system when at equilibrium obeys the Boltzmann distribution. Yakovenko used computer simulations to explain the inhomogeneity of the distribution of wealth, showing that the disparity between the rich and the poor is a natural phenomenon in the world. That is, the wealth distribution obeys the Pareto principle, meaning 20% of people enjoy 80% of the wealth and resources[10,11]. From the point of view of physics, entropy is a disordered state of molecular motion. From a statistical physics point of view, entropy is the logarithm of all particle states. While from the perspective of information, entropy is a measurement method of information. Few researchers analysed the development of the economy on the base of input-output tables using information entropy. Various methods are used, some of which involve to take into account of multiple components–internal and external subsystems and monitoring the entropy of complex systems, as reflected in the works of Vladimir Bulygin, Gennadiy Averin, Vladimir Opritov and Alexander Banaru[12,13]. Nevertheless, it is research gap in case of input-output data analyzing by use of information entropy. Evaluation of systems using entropy are developing branch of analysis. Therefore, this paper tasks to:

1. Analyse the efficiency and resilience (potential capacity) of the Chinese economy from the point of view of a complex economy and analyze the entropy of the internal provinces and industries subsystems. Describe how parameters of efficiency and resilience reflect the economic behavior;
2. Apply this approach to identify China Economy imbalance and prevented the most inadequate situation;
3. By using of spatial characteristics and spatial pattern of the industrial structure, analyze the basic point of the supply-side structural reform in China.

In the first Chapter of the Paper it is theoretical background literature review concerning complex science researches and information entropy background. The second part represents sample and data and methodological background, modeling formula. Third part is the obtaining results presentation concerning Province economy modeling and industrial economy modeling.

## Material and methods

### Complex Science development

Karl Ludwig von Bertalanffy (1901–1972) was an Austrian biologist known as one of the founders of general systems theory (GST)[14]. While the "conceptual part" of which was first introduced by Alexander Bogdanov. An essential reference work in almost all the Soviet published literature on the subject of system theory is Bogdanov’s (Alexander A. Malinovskii’s) three volume treatise, Tektologiya: Vseobshaya Organizacionnaya Nauka, in English named Tektology: The Universal Organizational Science)- in short namely Tektology, or “science of construction”, published in Russia during the period from 1912 to 1927[15]. The central thesis of Bogdanov’s Tektology is that there exist in nature and society an absolute unity in organizational methods that can be studied scientifically. Bogdanov proposed that the world is of dynamic changes, only the differences in energy tensions result in actions and reactions and only these differences have a practical meaning [16]. Tektology (sometimes transliterated as “tectology”) is a term used by Bogdanov to describe a discipline that consisted of unifying all social, biological and physical sciences by considering them as systems of relationships and by seeking the organizational principles that underlie all systems. Tectology is now regarded as a precursor of systems theory and related aspects of synergetics[16–19]. Ernst T. Haeckel developed the word “tectology”, but Bogdanov used it for a different purpose. His work about tekcology “Universal Organization Science” anticipated many of the ideas that were popularized later by Norbert Wiener in Cybernetics and Ludwig von Bertalanffy in the General Systems Theory. There are suggestions that both Wiener and von Bertalanffy might have read the German edition of Tekcology which was published in 1928[20].

According to Bogdanov “the aim of Tectology is the systematization of organized experience”, through the identification of universal organizational principles: “all things are organizational, all complexes could only be understood through their organizational characteristics. This is historically the first identification of philosophical “complexes” in the natural sciences to denote a combination of elements of “activity-resistance”. Bogdanov considered that any complexes should correspond to its environment and adapt to it, which nowadays is referred to in ecological economics. A “complex” is not identical to a “complicated, a hard-to-comprehend, large unit”. A stable and organized complex is greater than the sum of its parts. In Tectology, the term “stability” refers not to dynamic stability, but to the possibility of preserving the complex in the given environment [17].

In Tectology, Bogdanov made the first “modern” attempt to formulate the most general laws of organization. Tectology addressed issues such as holistic, emergent phenomena and systemic development. Tektology as a constructive science-built element into a functional entity uses general laws of organization. Bogdanov’s does not recognized differences between observation and perception according to his “empirio-monistic” (empirical in English) principle (1899), and thus he created the beginning of a general empirical, trans-disciplinary science of physical organization, as an expedient unity and precursor of Systems Theory and Holism[19].

The “whole” in Tectology, and the laws of its integrity, were derived from biological rather than the physical view of the world. Regarding the three scientific cycles which comprise the basis of Tectology (mathematical, physical-biological, and natural-philosophical), it is from the physical-biological cycle that the central concepts have been taken and universalized [17].

The starting point in Bogdanov’s Universal Science of the Organization—Tectology (1913–1922) was that nature has a general, organized character, with one set of laws of organization for all objects. This set of laws also organizes the internal development of the complex units, as implied by Simona Poustilnik’s “macro-paradigm”, which induces synergistic consequences into an adaptive assembling phenomenon (1995). Bogdanov’s visionary view of nature was one of an ‘organization’ with interconnected systems. Bogdanov’s Tectology outlined the concepts and concerns of Complexity Theory a full 50 years in advance of chaos and fractal mathematics. In the research authors base on the Systems Economics principles. If someone were to analyse contemporary notions and fashionable catchwords, he would find “systems” high on the list. The roots of any development are complex one. Systems Theory focuses on the relations between the parts. Rather than reducing an entity such as the human body into its parts or elements (e.g., organs or cells), systems theory focuses on the arrangement of and relations between the parts and how they work together as a whole. This point of view is often referred to as a holistic approach to understanding phenomena. Flood and Jackson (1991) define a system as a complex and highly interlinked network of parts exhibiting synergistic properties-the whole is greater than the sum of its parts [21]. It is a collection of interrelated parts acting together to achieve some goal which exists in the environment. Also, the system is defined as a set of objects together with relationships between the objects and between their attributes related to each other and the environment to create or form a whole. Further, in 1981 Checkland defines a system as a model of a whole entity, which may apply to the human activity [22]. Accordingly, the actual problem of the modern economic theory is finding such a paradigm that could reflect economic processes taking place in the objective reality with a high degree of reliability [23]. The system paradigm introduced into scientific practice by J. Kornai from Harvard University in 1998 satisfied these conditions, which is complemented with other well-known economic paradigms, such as the neoclassical, institutional, evolutionary [24]. The main attributes of the system paradigm [24][25][26] is that “researchers who think regarding of the system paradigm are concerned with the system as a whole, and with the relations between the whole and its parts.” Russian scientist and corresponding Member of the Russian Academy of Science, George Kleiner, developed this idea. Kleiner claimed that fundamental aspects of the problem of economic sustainability must be considered from the position of the system economic theory and defined the potential of the system economic theory in the field of space-time analysis[22,27].

### Information entropy background

Count Carnot (1753–1823), a French mathematician and engineer, published the paper “Fundamental Principles of Equilibrium and Movement” in 1803. He supported that it is an impossibility of perpetual motion, due to “energy lost” during operation[28]. Another French physicist and engineer named Sadi Carnot (1796–1832), idealized a “heat engine”, showed that the thermal efficiency of a heat engine depends on temperature of source and sink[29]. German physicist and mathematician Rudolf Clausius (1822–1888), resolved problems of Sadi Carnot’s work during the year 1850 to 1860. In 1865, Clausius defined entropy, meaning “transformative content of energy” and “the entropy is of the universe tends to a maximum”[30,31]. Ludwig Boltzmann (1844–1906), an Austrian physicist and philosopher, published papers on kinetic theory of gases from the year 1872 to 1906. He created the logarithmic connection between probability theory and thermodynamic entropy and made entropy application to economics world possible[32]. American mathematician, engineer and cryptographer -Claude Shannon (1916–2001), introduced the concept of information entropy in his 1948 paper, named “A Mathematical Theory of Communication”. Claude Shannon independently discovered entropy in flows of information and invented “information theory”. The Mutual information is a concept rooted in information theory, which was introduced by Claude Shannon as the rate of transmission in 1948. He also introduced the entropy concept for a single discrete variable, and defined the joint entropy and conditional entropy for two discrete variables using the joint distribution. He then defined the rate of transmission as the difference between the entropy and the conditional entropy[33]. Later, American mathematician Edwin Thompson Jaynes (1922–1998), showed that the concept of entropy applies very broadly and Thermodynamic entropy is just one example[34–36].

Classical physics attempts to analyze all single objects, such as molecules, using Newton's laws. Thermodynamics, on the other hand, deals only with macroscopic phenomena. The laws between energy and entropy, called the microscopic states of molecules. According to Boltzmann's entropy theory, the occurrence frequency of N individuals (referred to as agents) in the system is at most N/2, and occasionally reaches the number of attachments of N/2 plus or minus 1. The possibility of occurring in the system, as formula Ω (k)

$${\Omega (k)=C}_{N}^{k}=\frac{N!}{k!(N-k)!}=\frac{N!}{N_{1}{!N}_{2}{!\ldots N}_{k}!}$$ (1)

Where, N is the number of individuals (referred to as agents). K is the total number of possibilities in the system

While Statistical physics relates microstates to macro-states, the origin of the Boltzmann entropy formula [37], expressed as follows:

S=klogW (2)

Where, S is the entropy of the system, K is the Boltzmann constant, and W is the number of microscopic states in a given macroscopic state. And the probability theory expression is used to measure the uncertainty of the result A concluding paragraph in this section can clarify the research contributions based on the identified gaps in the literatures. The authors are highly recommended to take some time and prepare a well-structured table for the review of differential and other theoretic models. Please pay attention to the fields select for this abstract review in Table 1.

**Table 1:** Literature review on system science.

Research Area	Author	Main Mind	Year
Complex Systems	Alexander Bogdanov	Tektology: The Universal Organizational Science	1898–1927
	Andrey Kolmogorov	One of the founders in the modern theory of probability, topology, geometry, mathematical logic, turbulence theory, the algorithm complexity theory, information theory, function theory, the dynamic system theory, functional analysis and several other areas of mathematics and its application	1950
	Karl Ludwig von Bertalanffy	General Systems Theory	1968
	Peter Checkland	Outlines the components of Soft Systems Methodology (SSM). Defines a system as a model of a whole entity	1981
	Janos Kornai	The system paradigm introduced into scientific practice	1981
	George Kleiner	Tetrade system economics model	1998
	David Rousseau	General Systems Theory: Its Present and Potential	2015
	Chikere Cornell C and Nwoka Jude	The Systems Theory of Management in Modern Day Organizations—A Study of Aldgate Congress Resort Limited Port Harcourt	2015
Information Entropy	Rudolf Clausius	Defined entropy, meaning “transformative content of energy” and “the entropy is of the universe tends to a maximum”.	1850–1860
	Count Carnot	Fundamental Principles of Equilibrium and Movement	1803
	Sadi Carnot	Reflections on the Motive Power of Fire, thermal efficiency of a heat engine depends on temperature of source and sink.	1824
	Josiah Willard Gibbs	Improved on the idea of energy by including changes in entropy.	1865–1903
	Ludwig Boltzmann	Created the logarithmic connection between probability theory and thermodynamic entropy and made entropy application to economics world possible.	1872–1906
	Vladimir Bekhterev	23 universally valid l laws: law of conservation of energy, law of gravity, law of repulsion, inertia, entropy, continuous motion and variability	1857–1927
	Erwin Rudolf Joseph Aleksandr Schrödinger	The founder of quantum physics, studied thermodynamic fluctuations and related phenomena, published papers on statistical physics, on the nature of the second law of thermodynamics and the reversibility of the laws of physics in time, and on the direction of entropy increase	1944–1946
	Claude Shannon	A Mathematical Theory of Communication	1948
	Norbert Wiener	Cybernetics	1950
	Nicholas Georgescu—Roegen	The laws of entropy and the economic process	1986
	Edwin Thompson Jaynes	Showed that the concept of entropy applies very broadly and Thermodynamic entropy is just one example.	1922–1998
	Vladimir Opritov	Biosystems entropy theory	1999
	Wang Hengjun	Advocated applying the concepts of energy and entropy in physics into economics and put forward the concepts of economic energy, economic entropy, absolute economic entropy, and relative economy.	2002
	Sergei Yakovenko	Creatively introduced the entropy principle in the economic research field in 2000, pointing out that money is similar to the of conservation of energy.	2012
	Gennadiy Averin	On the principle of existence and the law of increase the entropy in the context of genelas system representations of system dynamics"	2015
	Vladimir Bulygin	Entropy and Life from the Logic point of view	2016
	Alexander Banaru	Infornation Entropy of Feodorovs Groups	2018

### Data and main process

#### Data

The data from China's National Bureau of Statistics compiles national level input-output tables in open sources, published every five years since 1992. Research data is based on the public inter-provincial input-output tables of China, from the Division of National Economic Accounting department, National Bureau of Statistics. The authors model use China regional input and output table from historical point of view. Research has been done on the base of China’s 31 provinces and through 42 industrial sectors in 2012, while in 2007 the table only contains 30 provinces (without Tibet). According to the National Bureau of Statistics information, the 2017 table will be published in the end of 2019 or at the beginning of 2020. Therefore, the authors are modeling the China Economy from a historical point of view of 2007 and 2012.

The transformation of energy in a system is usually a discrete and unpredictable process. Probability Theory is an appropriate mathematical language for describing discrete events using statistical physics, where probability and logarithms can describe complex systems. Tribus believes that the system is continuously developing and changing, and the probability of its internal microstates is also constantly evolving, and the information can describe the reasons for the changes in the probability distribution[38,39].

#### Main process

The process of the entire formula is based on the ecological network formula created by Ulanowicz etc.[40–45], and the derivation process is shown in **S1 Text**[46–48]. The concept of information entropy is used to scale the sustainable development capacity H of a single system:

$$H=-K\sum_{i,j}\frac{T_{ij}}{T}log\frac{T_{ij}}{T}$$ (3)

Where, T is the total flow in the system, Tij represents the flow between node i and node j, and H represents the information entropy. K represents the adjustment coefficient:

$$X=K\sum_{i,j}\frac{T_{ij}}{T}log\frac{T_{ij}T}{T_{i}T_{j}}$$ (4)

Where, T is the total flow in the system, Tij represents the flow between node i and node j, Ti. represents the input flow, T.j represents the output flow. X represents the common entropy and K represents the adjustment coefficient by the formula is expressed as:

$$\Psi =-K\sum_{i,j}\frac{T_{ij}}{T}log\frac{T_{ij}^{2}}{T_{i}T_{j}}$$ (5)

Where, T is the total flow in the system, Tij represents the flow between node i and node j, Ti. represents the input flow, T.j represents the output flow, Ψ represented condition entropy and K represents adjustment coefficient

According to the algorithm, the following conclusions can be drawn:

H=X+Ψ (6)

Here in the Eqs (1–6), when it was used log2, the results units was Bit, and when was use lg, the results units was Nat. In this paper, author use log2, so the unit of all results is Bit[49].

The modelling of Public Inter-Provincial Input-Output Tables in China process is as follows:

Step 1: Calculate the X, Ψ of every China’ Province (so the result is 31 X and 31 Ψ in 2012). Considering that Beijing, Tianjin……and other Provinces (totally 31 Provinces) separate subsystem the boundary of every province (It is a closed system of every Subsystem-Province).

**Fig 1:**
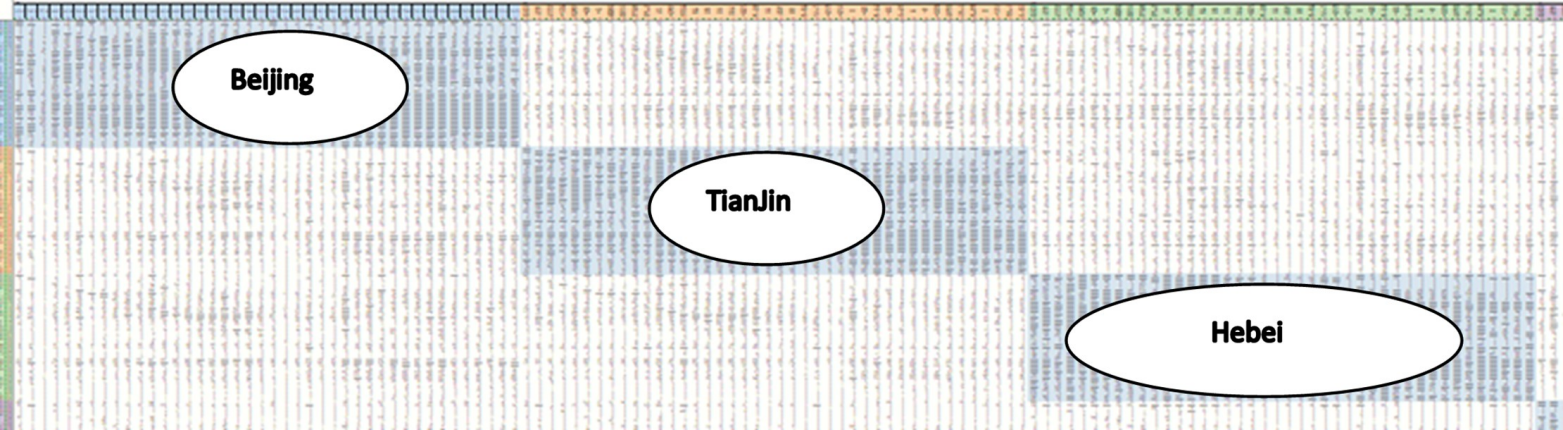
Regional Input-Output sheets sample.

**Fig 2:**
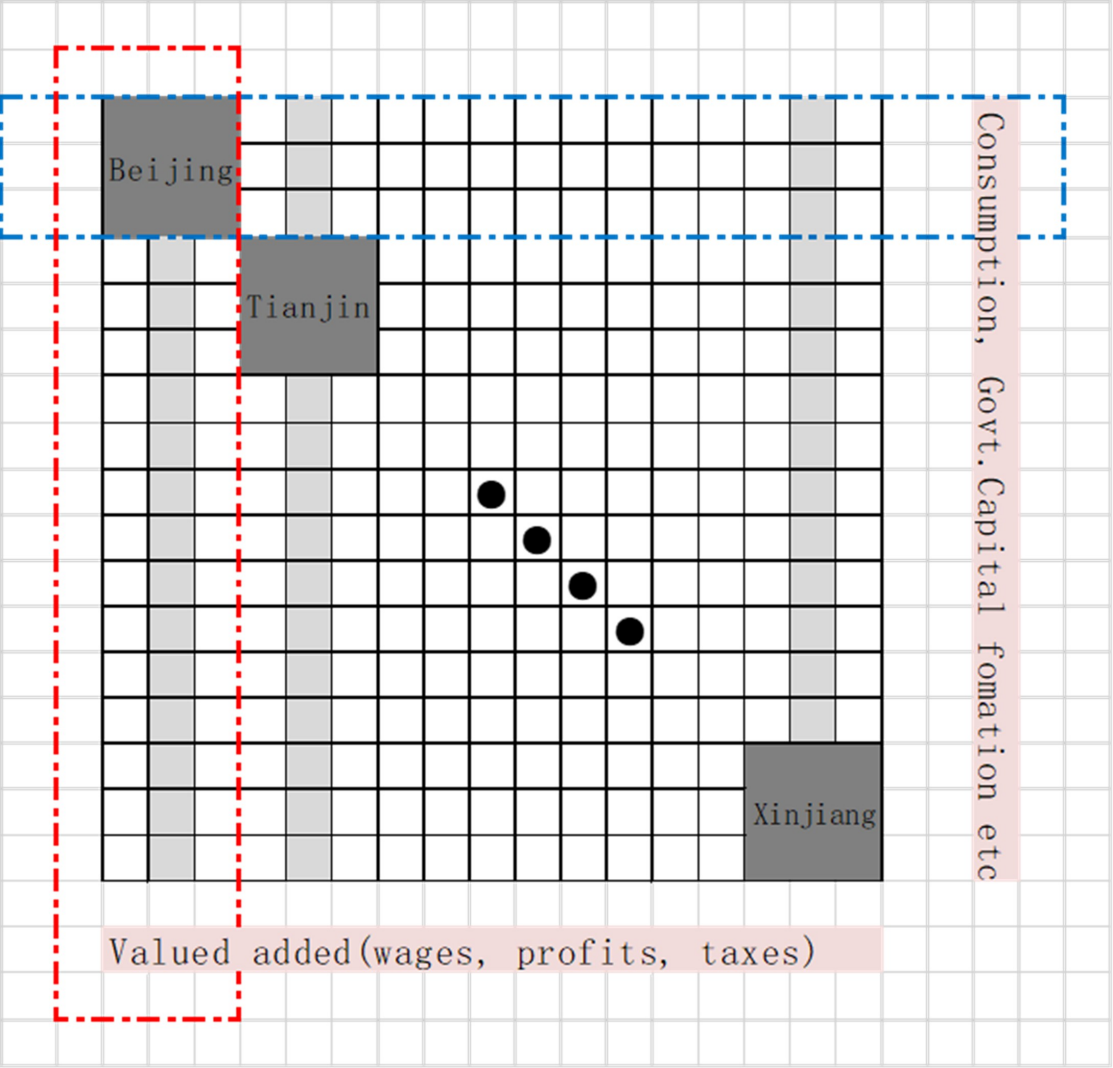
Boundary analysis (black areas–Provinces).

Step 2: Calculate input efficiency and potential growth which mean inputs is playing essential role to the one Inters Province. For example, the Authors ranked the transfers of products between provinces (thirty-one provinces multiply forty-two sectors input) to Beijing (or other Provinces). At the same time, what is the devotion of Beijing (42 sectors) to other Provinces (31 provinces *42sectors input). **Fig 1** above shows the miniature map of the China regional input-output table. In 2012 it has 31 provinces input and output and every province own 42 industrial sectors. Authors summarize the whole analysis, made abstraction modeling logistics and track in **Fig 2.**

In Fig 2, Every grey square represents one regional province. While every regional contains 42 sectors. The red dot rectangle means the national level output (for example, Beijing’s output devotes to other provinces), and the blue dot rectangle mean the national level input (for example, other province’s devotion to Beijing) (see red and blue dot of the boundary at the **Fig 2** Grouping by columns means input, and grouping by row mean output. The pink rectangles are the national level value added and national level final consumptions. And the black dots are on behalf of provinces. Thus, Fig 1 and Fig 2 are the same and Fig 2 is the sand table model.

### Results and analysis

#### Province economy modeling results

Based on the methodology of Step 1, Authors use Python 3.4 to program flows between provinces. Denoted that Beijing, Tianjin……and other provinces, (totally thirty-one provinces) are particular subsystems. From perspective of Province economy, Authors ranked valued of province in 2007 and 2012 and compared modeling results. In a closed system, authors calculate every province and find that all regions have value of efficiency near 1.5 and value of potential near 5.8 with very small difference. But in an open system (considering other province input and output), the results are quite different.

**Fig 3:**
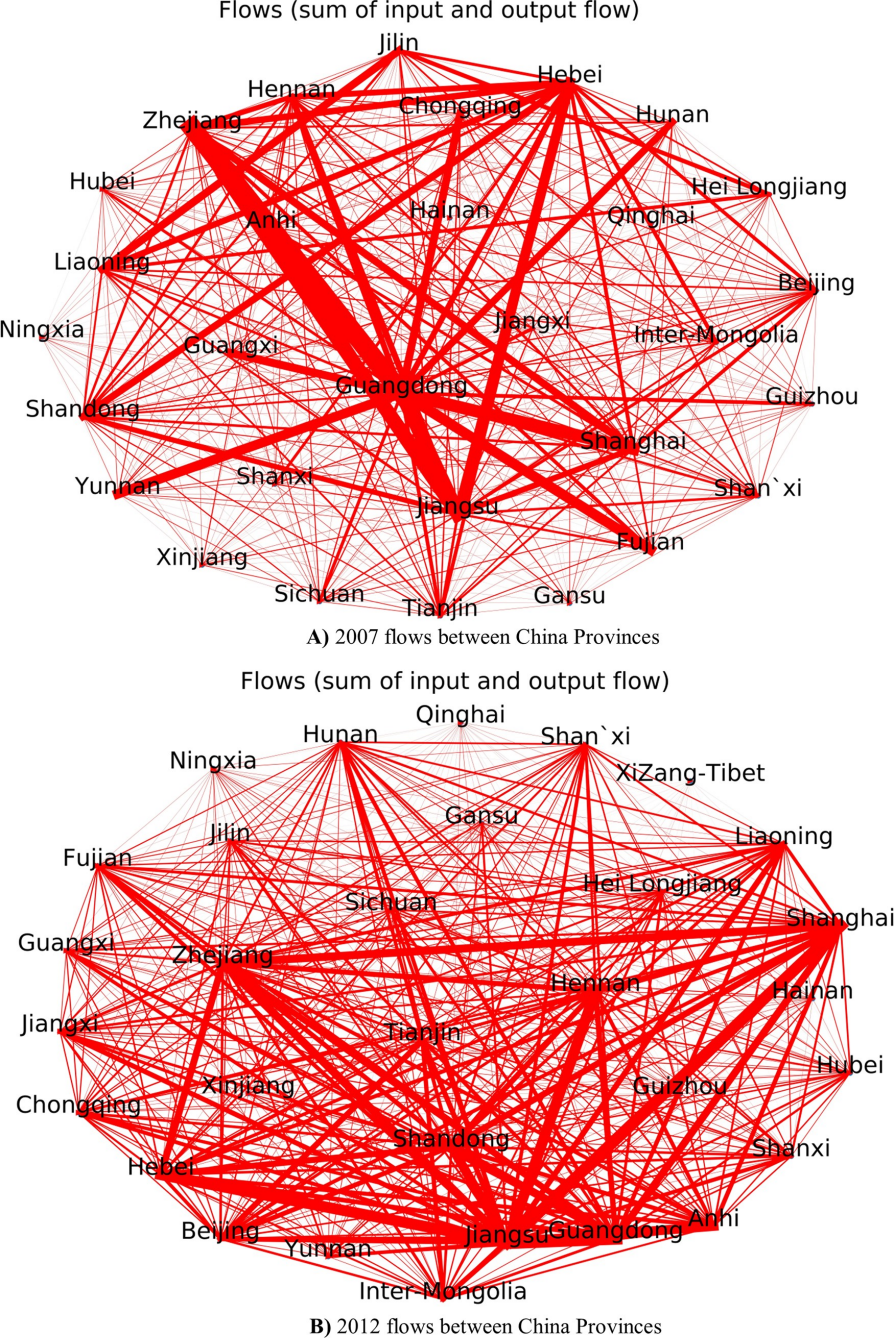
**A)** 2007 flows between China Provinces **B)** 2012 flows between China Provinces.

**Fig 3** shows the flows of input and output capital between Provinces. The largest flows are among Guangdong, Zhejiang, Jiangsu, Yunnan, Shanghai, Hebei, in 2007 as shown in **Fig 3A)**. While the biggest flows are among Guangdong, Zhejiang, Shandong, Hebei, Beijing, Tianjin, Inner Mongolia in 2012 as is shown in **Fig 3B).** Moreover, the flows illustrate the development of China’ inner-middle and inner-western provinces, such as Anhui, Hubei and Jiangxi.

**Fig 4:**
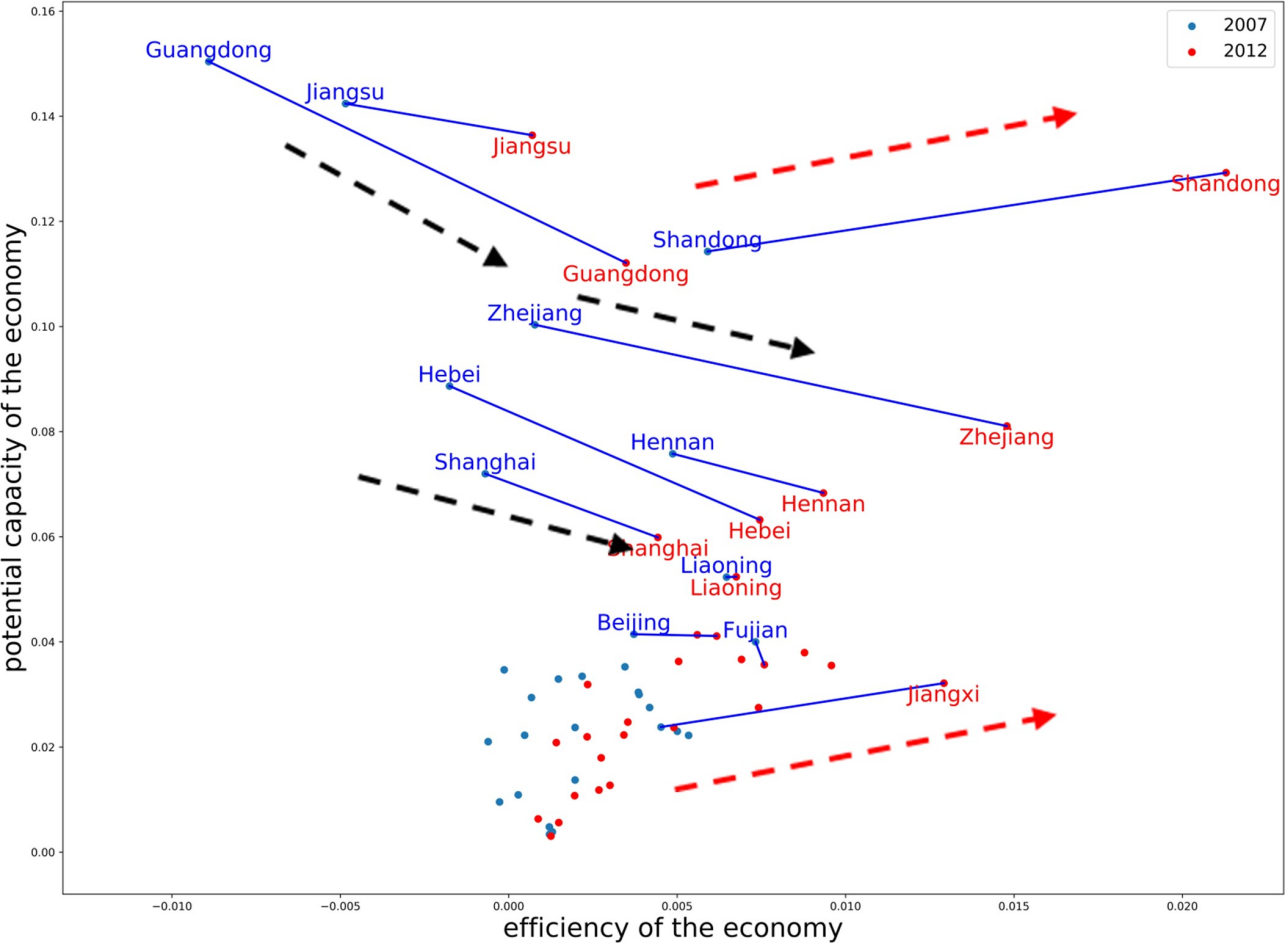
Efficiency and the potential of the province level in 2007 and 2012.

From the **Fig 4** plot, there are two trends observed in Provinces. Shandong Province had a big growth and other three provinces (Jiangxi, Hubei, Anhui) had a slight increase from 2007 to 2012 for both X and Psi values. At the same time, Jiangsu and Guangdong Psi had a big de-growth of value of phi.

Beijing and Liaoning had minor changes at the analyzed period. According to the development trends of the provincial economy and the study results, we can conclude that Shandong has the most economic potential among all the provinces. Other high economic potential provinces are Jiangxi, Hubei and Anhui.

Thus, the results of **Fig 3** and **Fig 4** has the consistency, that means when input-output flow increase, the X value increase. However, the trend of Ψ value is a little different. Guangdong has been the biggest producer of GDP for almost two decades, and Jiangsu, which rose strongly in the late 1990s, has been two of the most aggressive economic developed provinces. Although the three southern provinces Guangdong, Jiangsu and Zhejiang led China to benefits from opening and reform since 1987, their Ψ (potential economic capacity) value of those Provinces experienced relatively less growth from 2007 to 2012. T They are relatively less growth located eastern coastal areas and mainlyimport-export dependency. However, in 2012, Shandong’s input-output flow obvious increase, and both X and phi increase, that mean H has a big increase.

**Fig 5:**
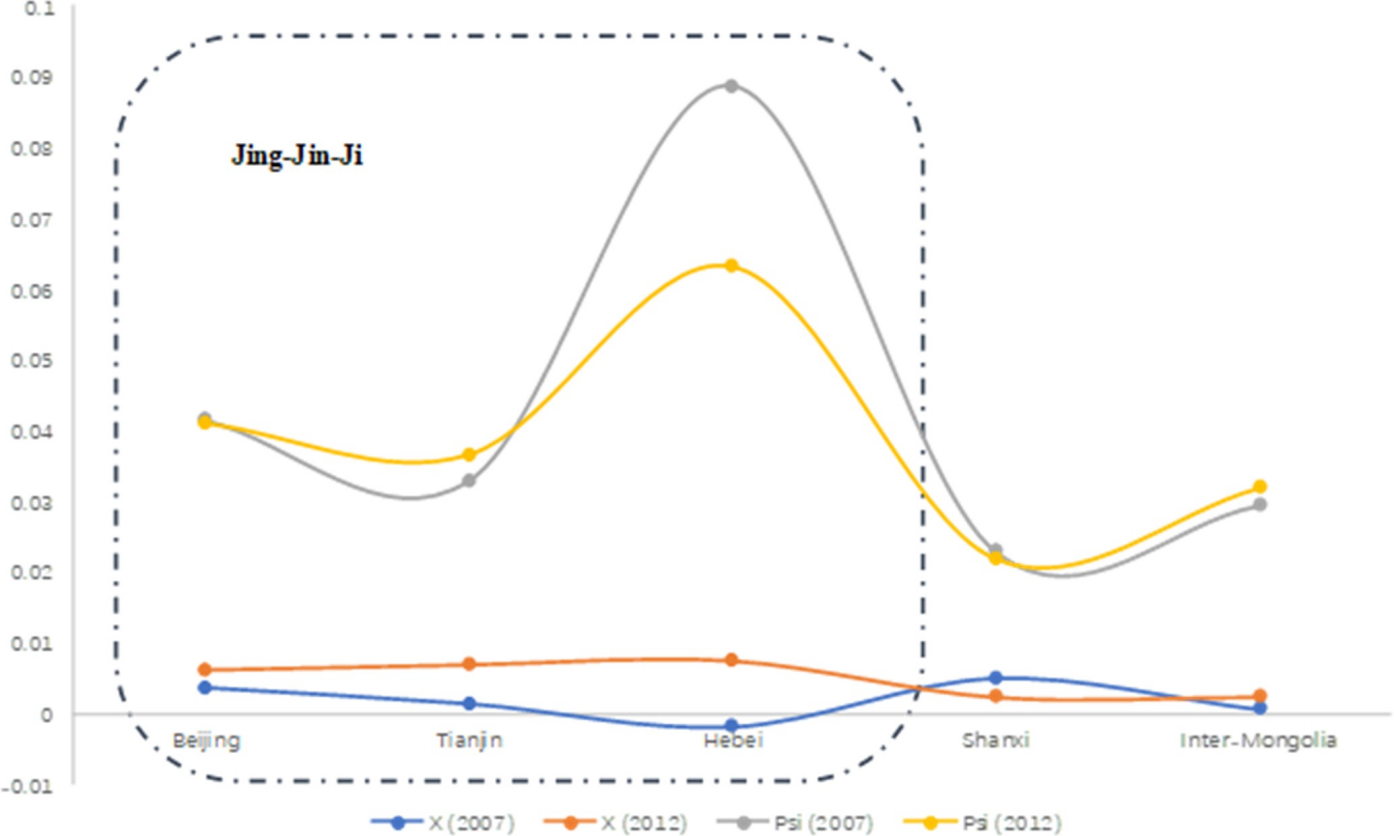
The value trend of X and Phi in Jing-Jin-Ji Regions 2007–2012.

Specially, the Beijing-Tianjin-Hebei region is the "capital circle" of China. Authors analyze Jing-jin-ji (Beijing-Tianjin-Hebei) region the trends of the X and Phi economics situation. This region takes the lead in interconnected development in China. From the Table 1 and **Fig 4** we can conclude that comparing the value of the three Beijing-Tianjin-Hebei Region, Hebei had a lower value of X in 2007, but Hebei had a big potential growth in 2007. The central government (CPC) implement a work report delivered on March 5, 2014, announced the plan is to strengthen economic cooperation in the Bohai rim region and the Beijing-Tianjin-Hebei region (Jing-jin-Ji) in order to unleash economic potential of Hebei. **Fig 5** shows that in 2007 as well as in 2012 the value among Jing-Jin-Ji, and most high potential capacity Regions is Hebei.

**Fig 6:**
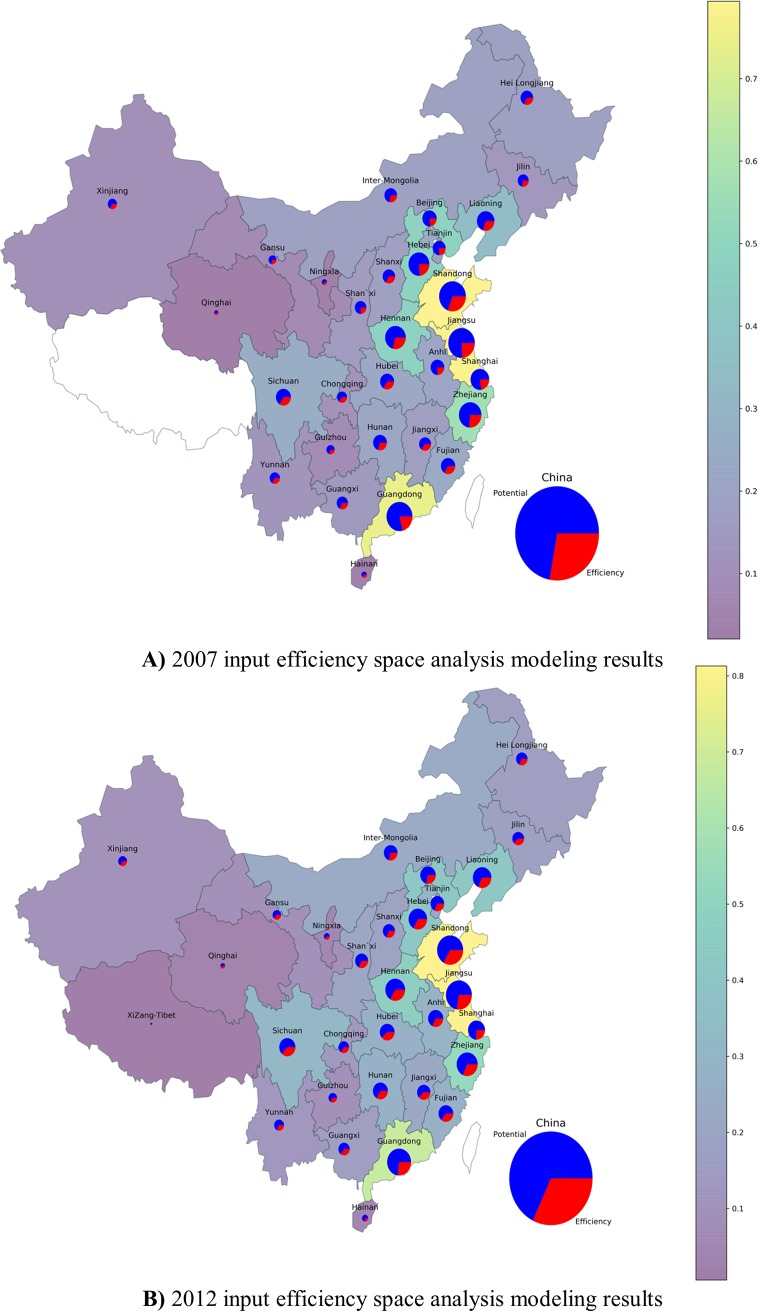
**A)** 2007 input efficiency space analysis modeling results. **B)** 2012 input efficiency space analysis modeling results.

Other provinces’ values of X and Ψ are shown in **SI 2.** According to the methodology of step 2, author calculate the provinces input efficiency and output efficiency, as well as input potential capacity and output potential capacity. Grouping by columns means input efficiency, and grouping by row mean output efficiency. **Fig 6A)** shows Guangdong, Jiangsu and Zhejiang had a big output efficiency in 2007. That mean Shandong, Henan, etc. provinces locate in the upstream of the industrial chain, mainly to provide production raw materials and Guangdong etc. provinces locate in the downstream of the industrial chain, mainly provide consumer products. Specially, in 2012, Shandong had a big growth both in input efficiency and output efficiency as is shown in **Fig 6B)**.

#### Industrial economy modeling results

**Fig 7:**
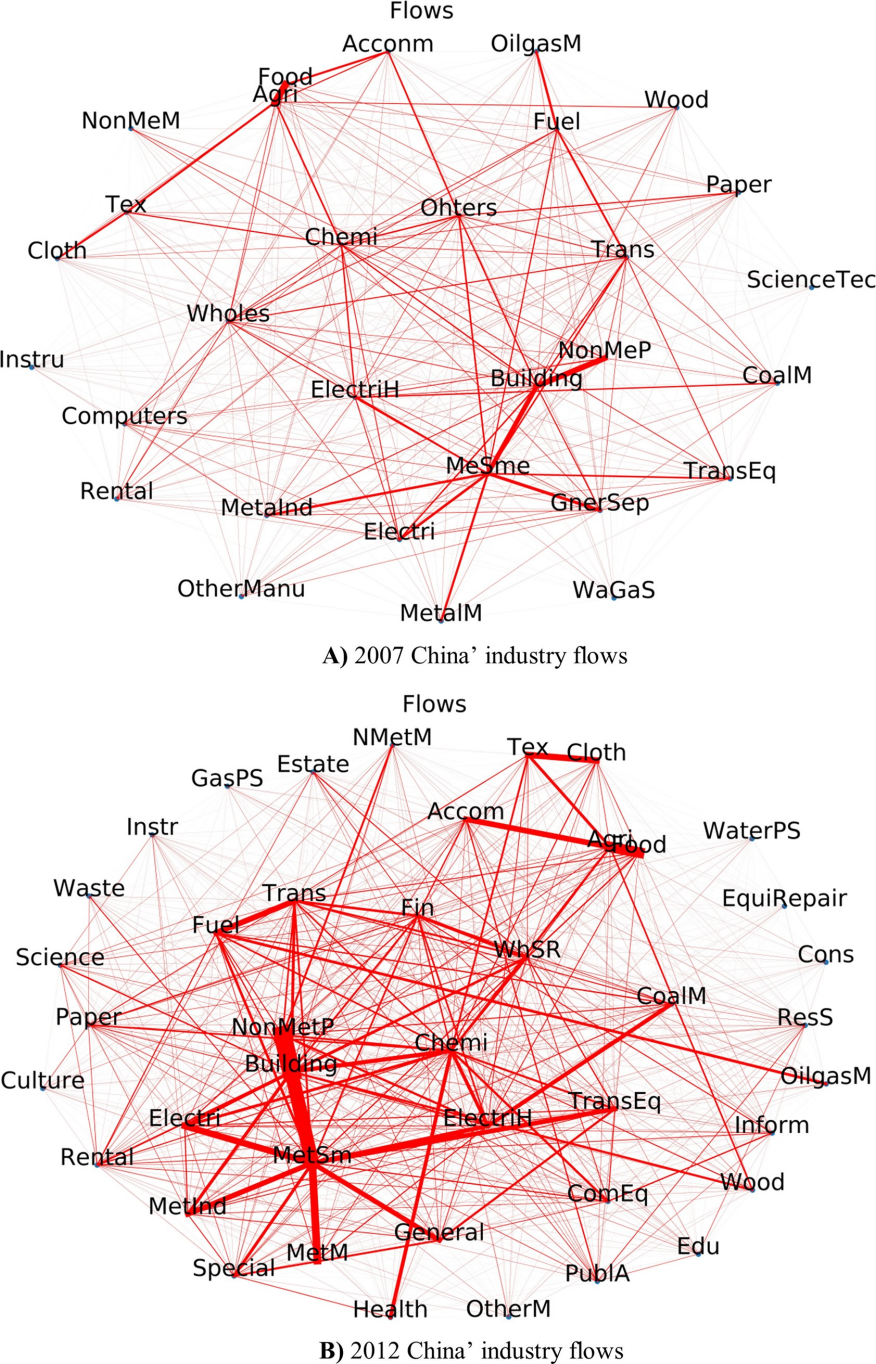
**A)**2007 China’ industry flows. **B)** 2012 China’ industry flows.

The biggest flows in 2007 are among the metal mining industry, metal smelting industry, metal industry and building industry (see **Fig 7A)**). While in 2012 there are nine big sectors: metal smelting, non-mental smelting, building, metal mining, metal industry, agriculture/forestry/animal husbandry/fishery products sector, food/tobacco sector, and oil and gas production sectors, petroleum/coking/nuclear fuel manufacture.

**Fig 8:**
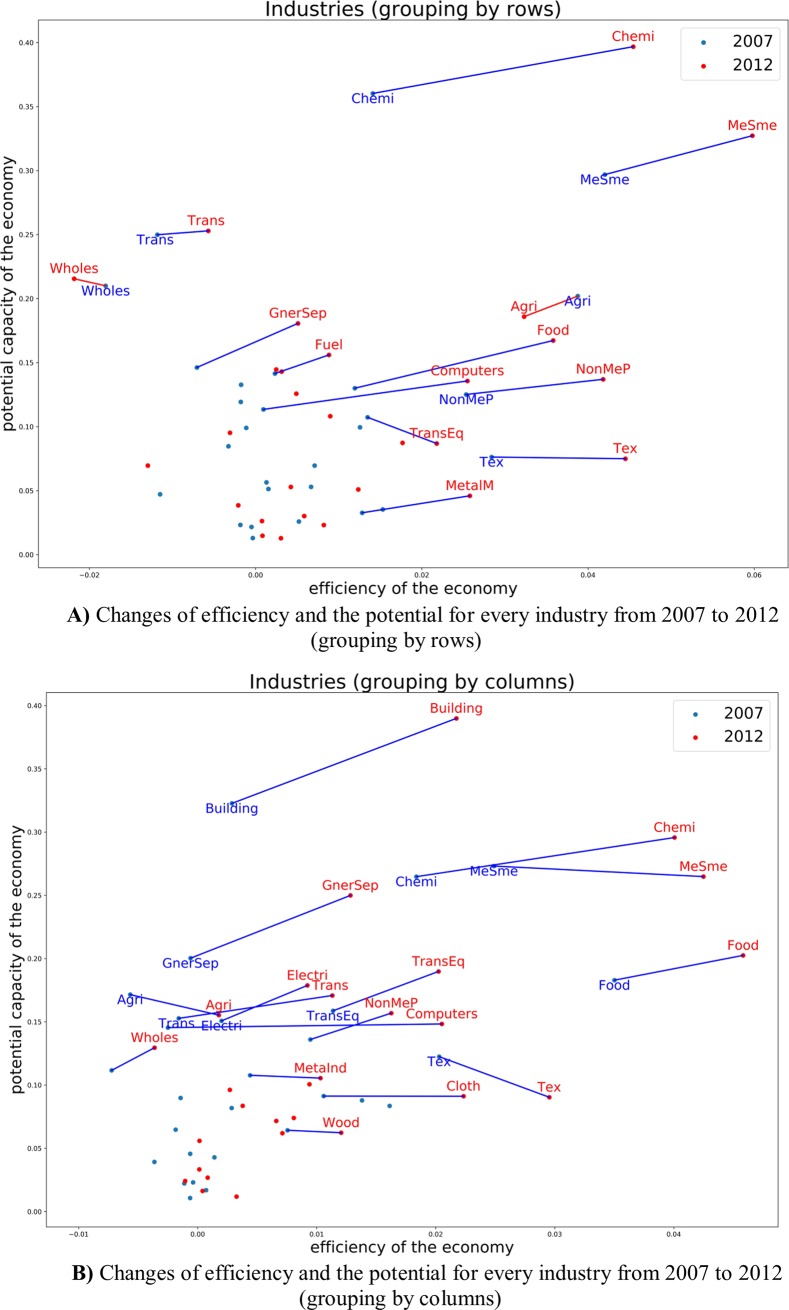
**A)** Efficiency and the potential of every industry in 2007 (grouping by rows and grouping by column). **B)** Efficiency and the potential of every industry in 2012 (grouping by rows and grouping by column).

Comparing the year 2007 and 2012, there are more flows in metal sector, mining-metal sector, smelting-metal sector and industry-building sectors and no-metal product -building sectors also began to play a vital role in the whole national economy. **Fig 8** reflects the vital sectors devoting to China economy in 2007 are building, metal products and no-mental products, general/special equipment, electrical machinery and equipment sectors. Five years later, we can observe more links between sectors. In 2012 the vital industries’ secotors are building, metal smelting, metal products, and non-metal smelting, metal mined, electrical machinery and equipment. The biggest three industry sectors devoting to China economy are building, metal smelting and non-metal smelting.

Clothing, food, building and transportation sectors are not only the people's livelihood, but also the internal driving force of China economic development. Thus, in 2012 there are big four flow chains as shown in **Fig 8**. Industry level of different provinces (industrial spatial layout) modeling results. We can observe every two branches, for example, thirty-one provinces multiply forty-two sectors input devotion to Beijing (or other Provinces). Beijing (forty-two sectors) devotion to other Provinces (thirty-one provinces multiply forty-two sectors).

The red line above shows the sectors’ mid-low value of potential growth such as transportation, wholesale and retailing, cloth, fuel and building sectors. That mean these are sunset industries. Especially, for fuel sector, both values of input and output of efficiency and potential growth had a downward trend. Computing and software sector had big growth in efficiency. **Fig 8A and 8B)** shows that the chemical industry sector and metal smelting sector are the two biggest industries in China both in 2007 and in 2012. While only ψ of the clothing sector decrease, that means clothing industry would have had potential growth from 2007 to 2012. Due to the different resource endowment and geographical location of each province, the industrial development is also different.

**Fig 9:**
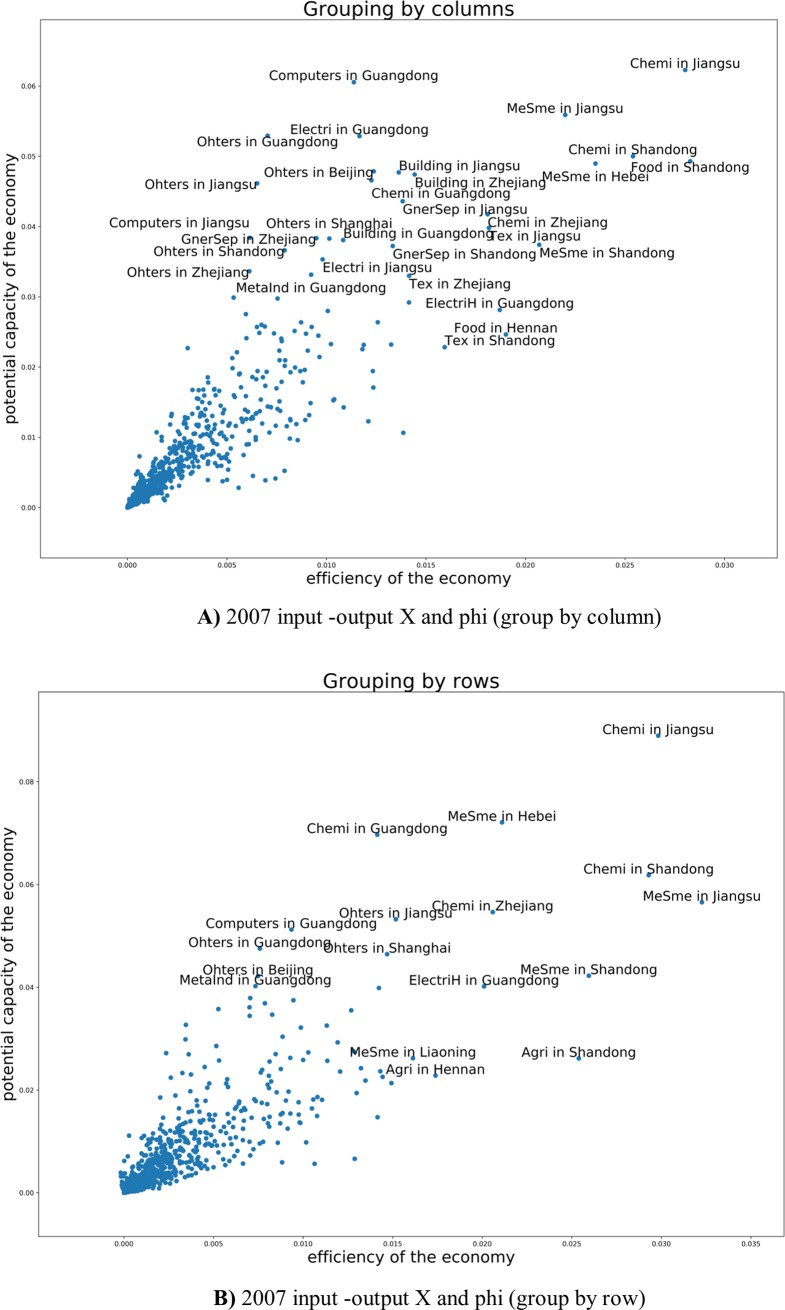
**A)** 2007 input -output X and phi (group by column). **B)** 2007 input -output X and phi (group by row).

**Fig 10:**
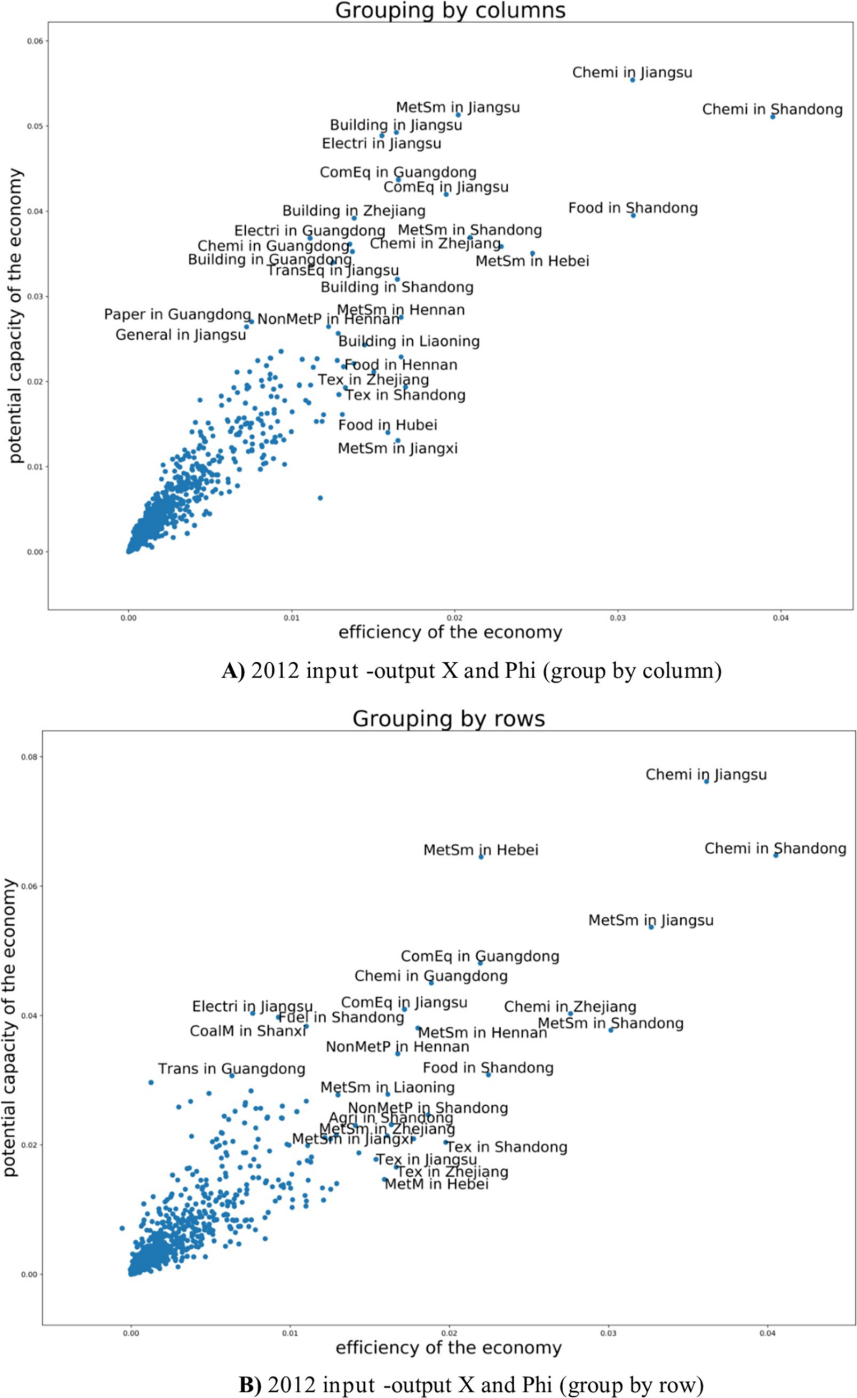
**A)** 2012 input -output X and Phi (group by column). **B)** 2012 input -output X and Phi (group by row).

**Fig 11:**
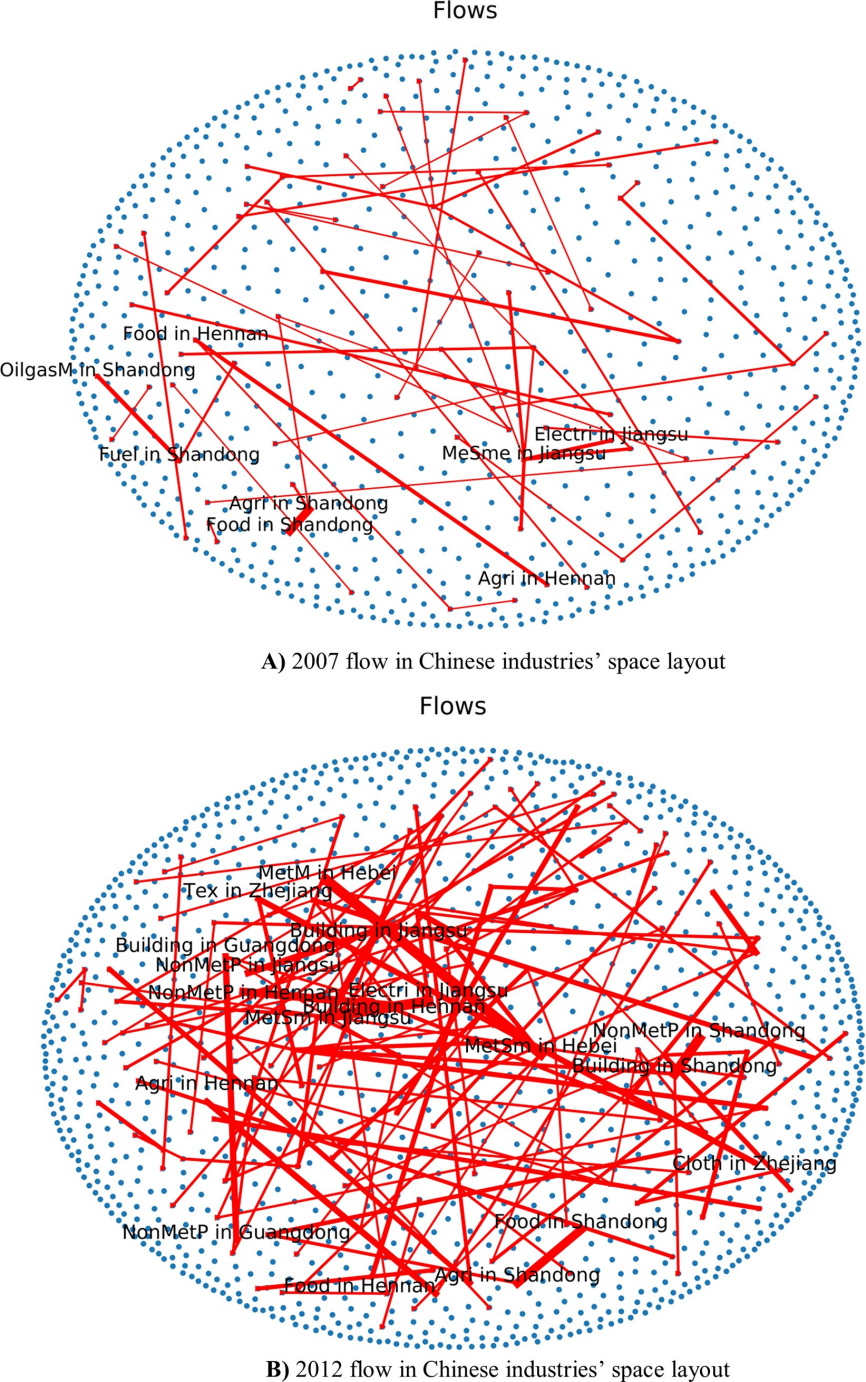
**A)** 2007 flow in Chinese industries’ space layout. **B)** 2012 flow in Chinese industries’ space layout.

As we can see from the **Fig 9A and 9B)** and **Fig 10A and 10B)**, Jiangsu province gave priority to the development of chemical and metal smelting industry. Shandong Province diversified industrial layout, gave priority to develop chemistry, metal industry and food sector. On the **Fig 11A and 11B)** we see the share of consumed resources (distributed of consumed resources).

From an industrial chain perspective, the most effective industry flows in Shandong province are from agriculture sector to the food sector. Other major flows of industry space layout are: from oil and gas mining sector to petroleum industry, from electricity to the metal smelting sector in Jiangsu Province and from agriculture sector to the food sector in Henan province based on the 2007 input-output table.

**Fig 12:**
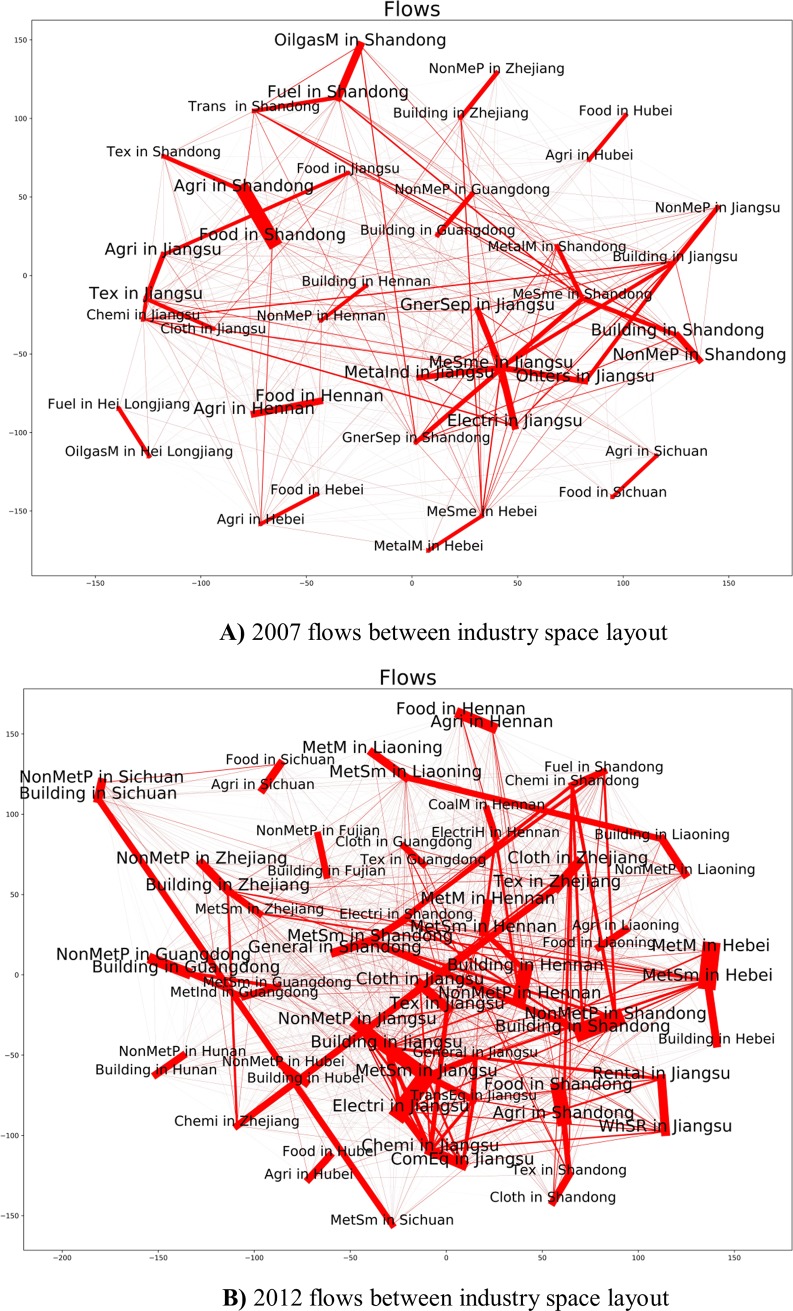
**A)** 2007 flows between industry space layout. **B)** 2012 flows between industry space layout.

In 2012 there are more flows between industries of different provinces, especially from metal and non-metal industry to building sectors in Henan and Shandong provinces as are shown in **Fig 12A and 12B).**

China's economy has a cluster character. Each region tries to produce and process its products to the end and consumes within the region. Each province has a cluster type, but most products are redistributed within a single cluster.

## Conclusion

The authors have arrived at conclusions from two angles and lines of logic: (1) from the perspective of provincial-level regions and (2) from the perspective of industries.

From the perspective of provincial-level regions the spatial imbalance of efficiency and potential of economic development was observed. The inequality in China’s regional development has been prominent from the 2007 and 2012 input-output table. From the modeling results, authors rank China’s provincial development in terms of input-output efficiency growth. The traditional perspective is to rank regional development only based on GDP growth, whereas here authors advocate a new evaluation method from efficiency and potential growth perspectives. Unbalanced regional economic development has become problematic and became a barrier for the national economy’s sustainability goals.

China’s regional economic policy had moved from fairness to efficiency since opening up and reform led by Deng Xiaoping. The eastern coastal provinces such as Guangdong, Jiangsu, Zhejiang Provinces took advantage of transportation, geography and policy advantages and became the leaders of economic development. However, there are still up and comers such as Shandong and Hebei Provinces with a great development potential.

From an industrial level’s perspectives, the results show that the industrial supply chain in China is too short, mainly focusing on the mining and processing of resources and minerals in the original upstream industry chain, while the downstream is not fully utilized. That is while, Understanding the spatial characteristics and spatial pattern of the industrial structure are the basic points of the supply-side structural reform in China. In practice people tend to carry out industrial policies and adjust the industrial structure under local province conditions. Industries related to people’s livelihoods including food, textile, building and transportation sectors had a big flow. However, chemical and metallurgical sectors still are the basic industries of national economy. After authors analyzed the efficiency and potential growth of China’ industrial structure between 2007 and 2012, they found that the distribution status and developing trend of different industries at the provincial level is essential. For example, chemistry in Jiangsu, information and software in Guangdong, agriculture and metal in Shandong make a big contribution to China economy.

However, a serious imbalance in the metal sector and no-mental sector industry chain and upstream resource sectors are indicating shortfalls. Meanwhile, the essential problem is the cheap export to foreign countries. Although China has a big market, the industry chain is too weak and too short. Take a non-melt industry for example, China exports major metals and nonmetallic primary minerals such as rare earths at much lower price, and then bought imported finished products at very high price. Compared with other countries, for example, Russia, there are mining-oriented regions, for example Kuzbas, and there are regions oriented to the final processing, for example Vologda[50,51]. For this reasons Russia has strong interregional flows, however, in China there are few longer value chain flows between Provinces. Especially China’s rare earth industry chain is very extreme as the high valued-added chain buys the end products from abroad at thousands of times of the price.

## Limitations and future research

The research has several constraints, which also sustain exciting avenues for future analysis. First, the China Economy input-output sheets, restricting the generalizability of research findings and limit the Study data to 2012 year. For further research, Authors will continue use China Economy input-output table 2017 year when available. Besides, 2007 Research data includes Tibet, 2012 –does not include such information. as Also, we only analyse 30 sectors in 2007, as consolidation structure had changed in 2012. All these factors can complicate the research. However, it is possible to overcome. As a future research direction, authors see the development of the Chinese economy on the basis of system science, space -time analysis, and information theory.

## Supporting information

S1 Text Formulation. (PDF)

S1 Table The values of X and ψ, grouping by column in 2007. (PDF)

S2 Table The values of X and ψ, grouping by row in 2007. (PDF)

S3 Table The values of X and ψ, grouping by column in 2012. (PDF)

S4 Table The values of X and ψ, grouping by row in 2012. (PDF)

S5 Table The values of X and ψ of industry level in 2007. (PDF)

S6 Table The Values of X and ψ of industry level in 2012. (PDF)

S7 Table List of acronyms. (PDF)
