# Correction: Complexity theory for the modern Chinese economy from an information entropy perspective: Modeling of economic efficiency and growth potential

**DOI:** 10.1371/journal.pone.0230165

**Published:** 2020-03-03

**Authors:** Jun Yan, Lianyong Feng, Artem Denisov, Alina Steblyanskaya, Jan-Pieter Oosterom

There is an error in affiliation 4 for author Alina Steblyanskaya. The correct affiliation 4 is: Laboratory of Microanalysis and Modeling, Central Economics and Mathematics Institute, Russian Academy of Science, Moscow, Russia.

In Fig 3, the Chinese providence “Shaanxi” is misspelled. The providence “Shan’xi” should be “Shaanxi.” Please see the correct Fig 3 here.

**Fig 3:**
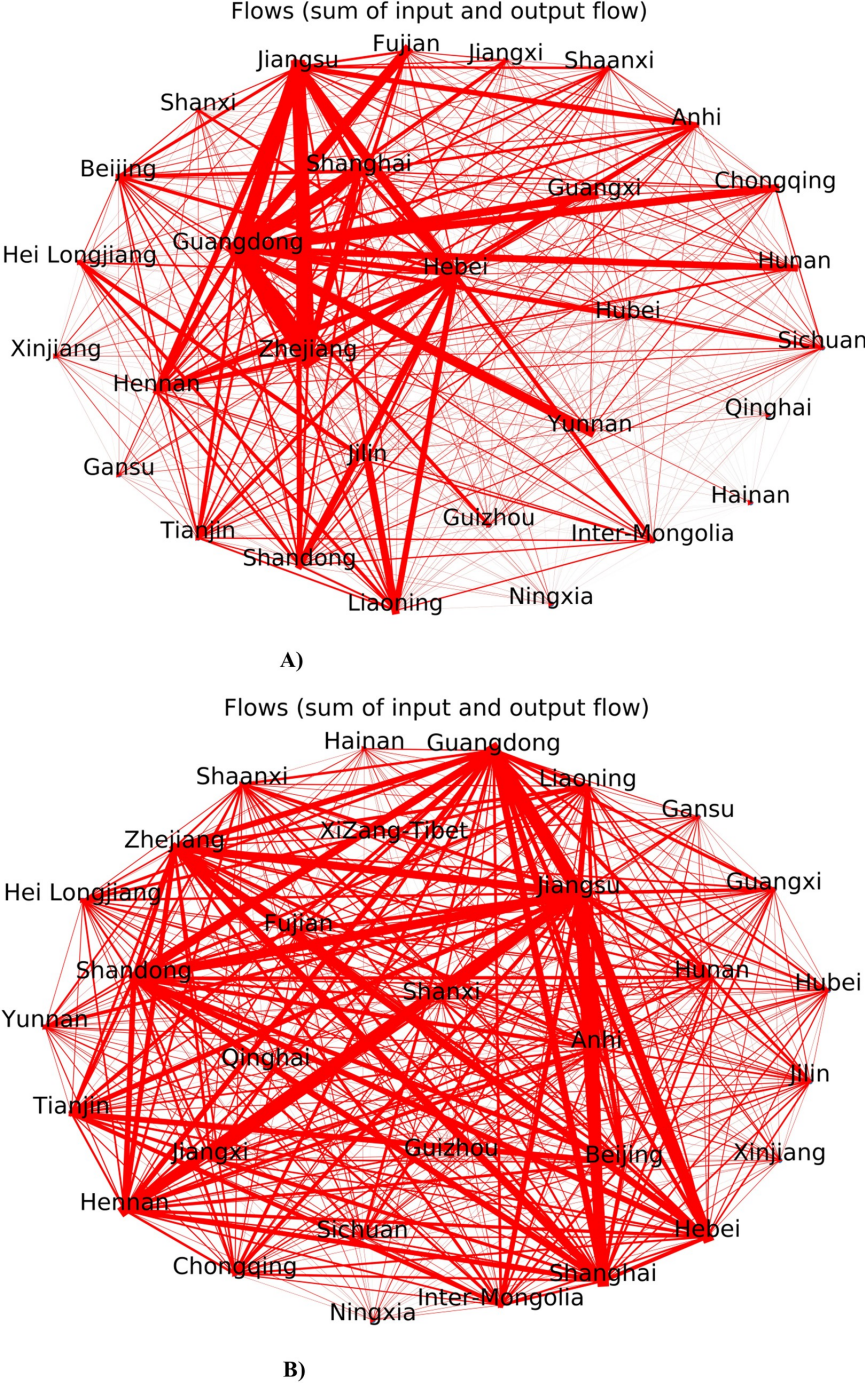
A) 2007 flows between China Provinces B) 2012 flows between China Provinces.
