# Supplementary material for: Complexity theory for the modern Chinese economy from an information entropy perspective: Modeling of economic efficiency and growth potential
Source: PLoS One. 2020 Jan 28;15(1):e0227206. doi: 10.1371/journal.pone.0227206 (PMC6986704; doi:10.1371/journal.pone.0227206)
Supplement: S1 Text — (PDF) [file pone.0227206.s001.pdf]

S 1 Text. Formulation

Despite Ulanowicz, Goerner, Lietaer, & Gomez (2009) made no direct mention to Shannon's work, the manuscript is all based on the work of other authors who derived their equations from Shannon's work[49–51].

Ulanowicz used the concept of flow in the ecological network to simulate the uncertainty of event results and found the inverse correlation of the probability of occurrence of the results. Therefore, the function expression as follows to measure the uncertainty of the result.

$$H_i = \mathrm{K}\log(\frac{1}{p_i}) \qquad (1)$$

or

$$H_i = -\mathrm{K}\log p_i \quad (2)$$

Where, H_i represents the uncertainty of result i, p_i represents the probability of result, and K is the proportionality constant.

For the uncertainty of all results in microstates, the weighting factor method is adopted to take the probability p_i of result I as the weighting factor, and the uncertainty of all results is weighted to calculate the uncertainty of all results.

$$\mathrm{H} = \sum_i p_i H_i \quad (3)$$

$$\mathrm{H} = -\mathrm{K}\sum_i p_i \log p_i \quad (4)$$

If the macroscopic state of the system is unknown, the equal probability event is usually adopted, and the hypothesis obeys the incomplete Bayesian effort principle. Then the average uncertainty formula is as follows, also known as the maximum entropy formula.

$$H_{max} = -\mathrm{K}\sum_i \frac{1}{n}\log\frac{1}{n} \qquad (5)$$

or

$$H_{max} = \mathrm{K}\log n \qquad (6)$$

H_{max} is the average maximum entropy (maximum uncertainty) of events without prior knowledge, and the posterior uncertainty is denoted as H. n is the number of macrostates in the system, they are equally likely events. K is the proportionality constant.

Where N is the information contained in m observations.

$$\mathrm{N} = H_{max} - H \qquad (7)$$

If the prior uncertainty is $\dot{p_i}$ and the posterior uncertainty is p_i, the reduction in information is N, which is expressed as follows:

$$N = -K(\log \dot{p}_i) - (-K \log p_i) \qquad (8)$$

or

$$N = K \ \log \frac{p_i}{\dot{p}_i} \ (9)$$

The reduction of the average uncertainty is understood as the average information obtained, denoted by N

$$N = K \ \sum p_i \log \frac{p_i}{\dot{p}_i} \qquad (10)$$

Here, based on previously defined in Probability theory, authors write out the joint probability and conditional probability.

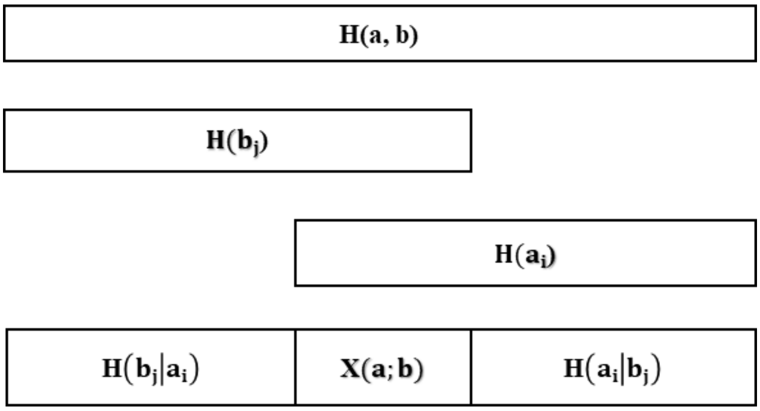

Figure, the relationship of joint probability and conditional probability

Conditional probability expressed as $p(b_i|a_j)$, and Joint probability expressed as $p(a_j, b_i)$, Joint probability refers to the probability that two different events occur simultaneously, the relation between them denoted as:

$$p(b_j|a_i) = p(a_i, b_j)/p(a_i) \quad (11)$$

$p(b_j|a_i)$ indicates the adjustment of the probability ofb_j given the probability of a_i next, Calculate the amount of information that b_j provides to a_i

$$[-K \log p(b_j)] - [-K \log(b_j|a_i)]$$

$$= K \log(b_j|a_i) - K \log p(b_j)$$

$$= K \log[\frac{p(b_j|a_i)}{p(b_j)}] \quad (12)$$

The average mutual information A of the whole system is denoted as

$$A = K \ \sum_{i,j} p(a_i, b_j) \log[\frac{p(b_j|a_i)}{p(b_j)}] \qquad (13)$$

Here, information theory is based on the entropy function. The average mutual

information can be used to quantify the connectivity or organization degree of the network in the system. This formula is applied to the input-output analysis of economic system. Where, a_i represents the energy input in the system, and b_j represents the output in the system. The total input in the system is denoted asT'_i， and the output in the system is denoted as T.

$$T'_i = \sum_{i=1}^{n} T_{ij} \quad (1)$$

$$T_j = \sum_{j=1}^{n} T_{ij} \qquad (15)$$

$$T=\sum_{j=1}^{n} T_j= \sum_{i=1}^{n} T'_i \qquad (16)$$

T is the total throughput in the system Here, authors establish links between T and p:

$$p(a_i) = \frac{T'_i}{T} \quad (17)$$

$$p(b_j) = \frac{T_j}{T} \quad (18)$$

Joint probability density:

$$p(a_i, b_j) = \frac{T_{ij}}{T} \quad (19)$$

Conditional probability density:

$$p(b_j|a_i) = \frac{p(a_i,j)}{p(a_i)} = \frac{T_{ij}}{T'_i} \qquad (20)$$

Average interactive information:

$$X(a; b) = K\sum_{j=1}^{j=n} \sum_{i=1}^{i=n} \frac{T_{ij}}{T} \log\frac{T_{ij}T}{T_j T'_i} \quad (21)$$

The Maximum of the whole system is denoted as:

$$H = -K\sum_{i,j} \frac{T_{ij}}{T} \log\frac{T_{ij}}{T} \quad (22)$$

Thus, the difference between the total uncertainty of the system H and the average mutual information X is the residual uncertainty of the system -- conditional entropy, which is denoted as Ψ:

$$\Psi=H-X \quad (23)$$
