# Supplementary material for: Complexity theory for the modern Chinese economy from an information entropy perspective: Modeling of economic efficiency and growth potential
Source: PLoS One. 2020 Jan 28;15(1):e0227206. doi: 10.1371/journal.pone.0227206 (PMC6986704; doi:10.1371/journal.pone.0227206)
Supplement: S1 Table — (PDF) [file pone.0227206.s002.pdf]

S1 Table. The values of X and ψ, grouping by column in 2007

X	Phi	Regions
0.06047060	0.18115058	**Beijing**
0.04676013	0.14212804	**Tianjin**
0.10588657	0.32790458	**Hebei**
0.05293165	0.09669836	**Shanxi**
0.04524874	0.09643176	**Inter-Mongolia**
0.10236755	0.21804182	**Liaoning**
0.04096112	0.10132109	**Jilin**
0.05361969	0.10826171	**Hei Longjiang**
0.09325433	0.30985154	**Shanghai**
0.19456800	0.61052693	**Jiangsu**
0.15954132	0.48401870	**Zhejiang**
0.05145781	0.15526334	**Anhi**
0.07683375	0.17104490	**Fujian**
0.05649758	0.10794613	**Jiangxi**
0.24178258	0.53713333	**Shandong**
0.13313397	0.29673776	**Hennan**
0.07213125	0.14779910	**Hubei**
0.06406245	0.14022997	**Hunan**
0.17238308	0.63169490	**Guangdong**
0.04479380	0.09280006	**Guangxi**
0.01431738	0.01753960	**Hainan**
0.04189358	0.08491996	**Chongqing**
0.08435220	0.17174201	**Sichuan**
0.02203858	0.05164772	**Guizhou**
0.03476156	0.08199653	**Yunnan**
0.03257321	0.10277266	**Shan`xi**
0.02489801	0.04138895	**Gansu**
0.00881747	0.01422557	**Qinghai**
0.01022606	0.01907686	**Ningxia**
0.02809780	0.05866788	**Xinjiang**
2.17066182	5.60096231	**Sum**
