# Supplementary material for: Complexity theory for the modern Chinese economy from an information entropy perspective: Modeling of economic efficiency and growth potential
Source: PLoS One. 2020 Jan 28;15(1):e0227206. doi: 10.1371/journal.pone.0227206 (PMC6986704; doi:10.1371/journal.pone.0227206)
Supplement: S2 Table — (PDF) [file pone.0227206.s003.pdf]

S2 Table. The values of X and ψ, grouping by row in 2007

X	phi	location
0.05321476	0.17505609	**Beijing**
0.04619486	0.13789510	**Tianjin**
0.11478402	0.36634713	**Hebei**
0.05839759	0.11537537	**Shanxi**
0.05096575	0.12653087	**Inter-Mongolia**
0.10245635	0.23884997	**Liaoning**
0.03998204	0.09519858	**Jilin**
0.05728765	0.12227788	**Hei Longjiang**
0.08752435	0.30070084	**Shanghai**
0.18949840	0.59433428	**Jiangsu**
0.14912235	0.41612591	**Zhejiang**
0.05262832	0.15414240	**Anhi**
0.07700552	0.15127403	**Fujian**
0.05751526	0.10315080	**Jiangxi**
0.24420287	0.54963099	**Shandong**
0.13962670	0.33394165	**Hennan**
0.07281731	0.14597096	**Hubei**
0.06404416	0.14771645	**Hunan**
0.15734205	0.58765494	**Guangdong**
0.04647705	0.09706314	**Guangxi**
0.01377917	0.02036673	**Hainan**
0.03978561	0.07124985	**Chongqing**
0.08418224	0.16438193	**Sichuan**
0.02358229	0.05244331	**Guizhou**
0.03882551	0.07871823	**Yunnan**
0.03619193	0.11247890	**Shan`xi**
0.02119243	0.04836280	**Gansu**
0.00913465	0.01150619	**Qinghai**
0.01044291	0.01918785	**Ningxia**
0.03245771	0.06302915	**Xinjiang**
2.17066183	5.60096233	**Sum**
