# Supplementary material for: Complexity theory for the modern Chinese economy from an information entropy perspective: Modeling of economic efficiency and growth potential
Source: PLoS One. 2020 Jan 28;15(1):e0227206. doi: 10.1371/journal.pone.0227206 (PMC6986704; doi:10.1371/journal.pone.0227206)
Supplement: S3 Table — (PDF) [file pone.0227206.s004.pdf]

S3 Table. The values of X and ψ, grouping by column in 2012

X	Phi	Regions
0.08354939	0.18908795	**Beijing**
0.06932464	0.13720070	**Tianjin**
0.13284108	0.24596904	**Hebei**
0.05003935	0.10828094	**Shanxi**
0.06356712	0.13021861	**Inter-Mongolia**
0.13135882	0.28314068	**Liaoning**
0.06316982	0.11273534	**Jilin**
0.04594014	0.09989707	**Hei Longjiang**
0.09695947	0.24928308	**Shanghai**
0.23470085	0.59869994	**Jiangsu**
0.17473698	0.40179663	**Zhejiang**
0.08993134	0.20661549	**Anhi**
0.09407087	0.17631763	**Fujian**
0.08245804	0.13873607	**Jiangxi**
0.27516652	0.50757211	**Shandong**
0.15155856	0.31312140	**Hennan**
0.10166646	0.16762491	**Hubei**
0.09228288	0.17527507	**Hunan**
0.18913159	0.50213028	**Guangdong**
0.05208882	0.10052894	**Guangxi**
0.01309544	0.03182281	**Hainan**
0.04756083	0.10712252	**Chongqing**
0.11356234	0.21372720	**Sichuan**
0.02912327	0.05576736	**Guizhou**
0.03704736	0.09225567	**Yunnan**
0.00251067	0.00675224	**XiZang-Tibet**
0.06016963	0.12620209	**Shan`xi**
0.03303304	0.05441069	**Gansu**
0.01221331	0.01272990	**Qinghai**
0.01560087	0.02494197	**Ningxia**
0.02934052	0.06160816	**Xinjiang**
2.66780002	5.63157249	**Sum**
