# Supplementary material for: Complexity theory for the modern Chinese economy from an information entropy perspective: Modeling of economic efficiency and growth potential
Source: PLoS One. 2020 Jan 28;15(1):e0227206. doi: 10.1371/journal.pone.0227206 (PMC6986704; doi:10.1371/journal.pone.0227206)
Supplement: S4 Table — (PDF) [file pone.0227206.s005.pdf]

**S4 Table. The values of X and $\psi$, grouping by row in 2012**

| X | Phi | Regions |
|---|---|---|
| 0.08279025 | 0.20430722 | **Beijing** |
| 0.07210351 | 0.15143567 | **Tianjin** |
| 0.13413209 | 0.27576395 | **Hebei** |
| 0.05602685 | 0.12170958 | **Shanxi** |
| 0.06688016 | 0.15840111 | **Inter-Mongolia** |
| 0.13197873 | 0.28013673 | **Liaoning** |
| 0.06477160 | 0.10246358 | **Jilin** |
| 0.05098133 | 0.10644821 | **Hei Longjiang** |
| 0.08948437 | 0.25713326 | **Shanghai** |
| 0.22317810 | 0.58320225 | **Jiangsu** |
| 0.16840650 | 0.36084638 | **Zhejiang** |
| 0.08959450 | 0.18641716 | **Anhui** |
| 0.09385608 | 0.16745039 | **Fujian** |
| 0.08198550 | 0.13238287 | **Jiangxi** |
| 0.27257834 | 0.54038379 | **Shandong** |
| 0.15500212 | 0.31238575 | **Henan** |
| 0.10160518 | 0.16052414 | **Hubei** |
| 0.09178754 | 0.17652868 | **Hunan** |
| 0.18379410 | 0.50186487 | **Guangdong** |
| 0.05290076 | 0.10024830 | **Guangxi** |
| 0.01308483 | 0.03200639 | **Hainan** |
| 0.04564607 | 0.08661437 | **Chongqing** |
| 0.11580875 | 0.19788661 | **Sichuan** |
| 0.02957741 | 0.05803428 | **Guizhou** |
| 0.03762824 | 0.08012239 | **Yunnan** |
| 0.00247308 | 0.00340188 | **XiZang-Tibet** |
| 0.06597775 | 0.13550286 | **Shanxi** |
| 0.03323352 | 0.05561177 | **Gansu** |
| 0.01242923 | 0.01448534 | **Qinghai** |
| 0.01531447 | 0.02614465 | **Ningxia** |
| 0.03278907 | 0.06172804 | **Xinjiang** |
| 2.66780002 | 5.63157249 | **Sum** |
