# Supplementary material for: Complexity theory for the modern Chinese economy from an information entropy perspective: Modeling of economic efficiency and growth potential
Source: PLoS One. 2020 Jan 28;15(1):e0227206. doi: 10.1371/journal.pone.0227206 (PMC6986704; doi:10.1371/journal.pone.0227206)
Supplement: S5 Table — (PDF) [file pone.0227206.s006.pdf]

S5 Table. The values of X and ψ of industry level in 2007

	X	Phi	Industries
**0**	0.038766	0.201946	Agriculture, forestry, animal husbandry and fi...
**1**	0.007095	0.069547	Coal mining products
**2**	0.0153	0.035288	Oil and gas production products
**3**	0.012815	0.032693	Metal mineral products
**4**	0.005205	0.025905	Non-metallic minerals and other mineral products
**5**	0.011939	0.130006	Food and tobacco
**6**	0.028399	0.076231	textile
**7**	0.001306	0.056447	Textile clothing shoes and hats leather down a...
**8**	0.006667	0.052943	Wood work and furniture
**9**	0.012552	0.099499	Papermaking, printing, culture, education and ...
**10**	0.002322	0.141441	Petroleum, coking products and nuclear fuel pr...
**11**	0.014103	0.360256	Chemical products
**12**	0.025331	0.125121	Non-metallic mineral products
**13**	0.042012	0.296859	Metal smelting and calendering products
**14**	-0.00179	0.11932	Metal products
**15**	-0.007065	0.146212	General and Special equipment
**16**	0.013467	0.107275	Transportation equipment
**17**	-0.001752	0.132695	Electrical machinery and equipment
**18**	0.000941	0.113471	Communications equipment, computers and other ...
**19**	-0.001831	0.023219	Instrument and meter
**20**	0.001542	0.051316	Other manufactured products
**21**	0.020391	0.2473	Electricity and heart of Production and supply
**22**	-0.000501	0.021679	Gas and water production and supply
**23**	-0.011492	0.047161	building
**24**	-0.011822	0.249886	Transportation, warehousing
**25**	-0.018043	0.209992	Wholesale and retail
**26**	-0.003271	0.084662	Accommodation and catering
**27**	-0.001125	0.09903	Rental and business services
**28**	-0.000342	0.012987	Scientific research and experiment
**29**	-0.058414	0.425601	Other service
