# Supplementary material for: Complexity theory for the modern Chinese economy from an information entropy perspective: Modeling of economic efficiency and growth potential
Source: PLoS One. 2020 Jan 28;15(1):e0227206. doi: 10.1371/journal.pone.0227206 (PMC6986704; doi:10.1371/journal.pone.0227206)
Supplement: S6 Table — (PDF) [file pone.0227206.s007.pdf]

S6 Table. The values of X and ψ of industry level in 2012

	X	Phi	Industries
**0**	0.032301	0.185911	Agriculture, forestry, animal husbandry and fi...
**1**	0.017675	0.087308	Coal mining products
**2**	0.008201	0.023123	Oil and gas production products
**3**	0.025772	0.045954	Metal mineral products
**4**	0.005845	0.030155	Non-metallic minerals and other mineral products
**5**	0.035808	0.167258	Food and tobacco
**6**	0.044492	0.074941	textile
**7**	0.004234	0.052891	Textile clothing shoes and hats leather down a...
**8**	0.012358	0.050933	Wood work and furniture
**9**	0.008979	0.108159	Papermaking, printing, culture, education and ...
**10**	0.008822	0.156009	Petroleum, coking products and nuclear fuel pr...
**11**	0.045433	0.396779	Chemical products
**12**	0.041817	0.136953	Non-metallic mineral products
**13**	0.059773	0.327268	Metal smelting and calendering products
**14**	0.0049	0.125702	Metal products
**15**	0.006649	0.114172	General equipment
**16**	-0.001552	0.066483	Special equipment
**17**	0.021794	0.086806	Transportation equipment
**18**	0.003134	0.142972	Electrical machinery and equipment
**19**	0.025488	0.13567	Communications equipment, computers and other ...
**20**	0.000758	0.026244	Instrument and meter
**21**	0.000821	0.014782	Other manufactured products
**22**	0.004933	0.020043	Scrap waste
**23**	0.000465	0.012962	Metal products, machinery and equipment repair...
**24**	0.018682	0.249559	Production and supply of electricity and heat
**25**	0.003044	0.012826	Gas production and supply
**26**	-0.000008	0.008658	Water production and supply
**27**	-0.012958	0.060893	building
**28**	-0.021838	0.215513	Wholesale and retail
**29**	-0.005692	0.252928	Transportation, warehousing and post
**30**	-0.003086	0.095174	Accommodation and catering
**31**	-0.000468	0.063044	Information transmission, software and informa...
**32**	0.00095	0.233973	financial
**33**	-0.002504	0.059358	The real estate
**34**	0.00248	0.14459	Rental and business services
**35**	-0.002089	0.038522	Scientific research and technical services
**36**	-0.000572	0.006132	Management of water conservancy, environment a...
**37**	-0.001306	0.059359	Resident services, repairs and other services
**38**	-0.001951	0.020437	education
**39**	-0.001446	0.008329	Health and social work

**40**	-0.000713	0.018605	Culture, sports and entertainment
**41**	-0.002772	0.016744	Public administration, social security and soc...
