# Supplementary material for: Complexity theory for the modern Chinese economy from an information entropy perspective: Modeling of economic efficiency and growth potential
Source: PLoS One. 2020 Jan 28;15(1):e0227206. doi: 10.1371/journal.pone.0227206 (PMC6986704; doi:10.1371/journal.pone.0227206)
Supplement: S7 Table — (PDF) [file pone.0227206.s008.pdf]

S7 Table. List of acronyms

Acronyms		secors
Agri	01	Agriculture, forestry, animal husbandry and fishery industry
CoalM	02	Coal mining industry
OilgasM	03	Oil and gas expoloration industry
MetalM	04	Metal mineral industry
NonMeM	05	Non-metallic minerals and other mineral industry
Food	06	Food and tobacco
Tex	07	Textile
Cloth	08	Textile clothing shoes and hats leather down and its products
Wood	09	Wood work and furniture
Paper	10	Papermaking, printing, culture, education and sporting goods
Fuel	11	Petroleum, coking products and nuclear fuel processed products
Chemi	12	Chemical industry
NonMeP	13	Non-metallic product
MeSmel	14	Metal smelting and calendering
MetaInd	15	Metal industry
General	16	General and Special industry
TransEq	17	Transportation industry
Electri	18	Electrical machinery and equipment
Computers	19	Communications equipment, computers and other electronic
Instru	20	Instrument and meter
OtherManu	21	Other manufactured
Waste	22	Scrap waste industry
Electri	23	Electricity and heart of Production and supply
GasSup	24	Gas production and supply
WaterSup	25	Water production and supply
Buliding	26	building
Trans	27	Transportation, warehousing
Post	28	Post
Inform	29	Information transmission, software and information technology
Wholes	30	Wholesale and retail
Acconm	31	Accommodation and catering
Fina	32	Financial industry
Estate	33	The real estate
Rental	34	Rental and business services
Sciense	35	Scientific research and experiment
Technical	36	Comprehensive Technical services
Facilities	37	Management of water conservancy, environment and public facilities
Rsident	38	Resident services, repairs and other services
Edu	39	Education
Health	40	Health,social security and social welfare

Entertain	41	Culture, sports and entertainment
Admin	42	Public administration, social organization
